# Onset of depression and anxiety among patients with gout after diagnosis: a population-based incident cohort study

**DOI:** 10.1186/s41927-022-00288-6

**Published:** 2022-10-03

**Authors:** Alyssa Howren, Eric C. Sayre, Hyon K. Choi, J. Antonio Avina-Zubieta, Kam Shojania, Jamie Y. Park, Mary A. De Vera

**Affiliations:** 1 Faculty of Pharmaceutical Sciences, University of British Columbia, 2405 Wesbrook Mall, Vancouver, BC V6T 1Z3 Canada; 2 Collaboration for Outcomes Research and Evaluation, Vancouver, BC Canada; 3 Arthritis Research Canada, Vancouver, BC Canada; 4 Division of Rheumatology, Allergy and Immunology, Department of Medicine, Massachusetts General Hospital, Harvard Medical School, Boston, MA USA; 5 Division of Rheumatology, Department of Medicine, University of British Columbia Faculty of Medicine, Vancouver, BC Canada; 6 Centre for Health Evaluation and Outcome Science, Vancouver, BC Canada

**Keywords:** Depression, Anxiety, Gout, Epidemiology, Mental health

## Abstract

**Background:**

Gout may be associated with an increased incidence of mental health disorders, however, published findings have been limited and inconsistent. Therefore, our objective was to conduct a population-based cohort study to evaluate the incidence of depression and anxiety after gout diagnosis.

**Methods:**

We used linked population-based administrative health data in British Columbia, Canada that includes information on demographics, outpatient visits, and inpatient visits from the period of January 1, 1990 to March 31, 2018. We assessed depression and anxiety using validated *International Classification of Diseases, 9th and 10th Revision* coding algorithms. We applied multivariable Cox proportional hazard models to evaluate incident depression and anxiety among patients with gout in comparison to non-gout controls, adjusting for age, sex, neighbourhood income quintile, residence, comorbidities, and health care utilization.

**Results:**

We included 157,426 incident cases of gout (60.2% male; mean age 57.1 years) and 157,426 non-gout controls (60.2% male; mean age 56.9 years). The incidence rate of depression among individuals with gout and non-gout controls was 12.9 (95% confidence interval [CI] 12.7–13.2) and 11.1 (95% CI 10.9–11.4) per 1000 person-years, respectively. The incidence rate of anxiety for those with gout was 5.4 (95% CI 5.3–5.5) per 1000 person-years and for non-gout controls was 4.6 (95% CI 4.4–4.7) per 1000 person-years. Individuals with gout had an increased onset of depression (adjusted hazard ratio [aHR], 1.08; 95% CI 1.05–1.11) and anxiety (aHR, 1.10; 95% CI 1.05–1.14) compared to non-gout controls.

**Conclusion:**

Our population-based study shows an increased incidence of depression and anxiety following gout diagnosis in comparison to non-gout controls. Findings suggest the importance of considering psychiatric impacts in addition to the physical impacts of gout.

**Supplementary Information:**

The online version contains supplementary material available at 10.1186/s41927-022-00288-6.

## Background

Gout is characterized by an elevated concentration of monosodium urate and is largely undertreated, with a population-based study suggesting that only 22% of individuals with gout are prescribed urate lowering therapies [1]. As a consequence, many experience repeated gout flares that negatively impact their physical and psychosocial functioning [2, 3]. A recent synthesis has described the psychological impact of gout, specifically finding that the prevalence of depression and anxiety ranges from 1.9–40% and to 3.8–10%, respectively [4]. Of note, a challenge with studies reporting prevalence estimates is that the occurrence of depression and anxiety relative to the diagnosis of gout is unclear. Studies on incident depression or anxiety after gout diagnosis are limited and inconsistent. A 2018 US study by Singh et al. [5] on the incidence of depression found that having gout is associated with a 42% increased the onset of incident depression among adults ≥ 65 years, as compared to individuals without gout. In contrast, a 2015 study conducted in the UK by Prior et al. [6] using primary care databases found no association between gout and the onset of either depression or anxiety. Given these inconsistent data which may be subject to selection bias, we used population-based administrative health data with complete information on all inpatient and outpatient visits to evaluate incident depression and anxiety among adults after diagnosis of gout.

## Methods

### Data source

We used individual-level, de-identified health services data for the entire population of British Columbia (BC) (estimated 5.0 million in 2018) [7], held by Population Data BC including: all outpatient visits (e.g., rheumatologists, general practitioners) in the Medical Services Plan (MSP) database [8], inpatient visits in the Discharge Abstract Database [9], deaths from Vital Statistics File [10], and demographics (e.g., age, sex) from the Consolidation File [11] from April 1985 onwards. Data were also linked from PharmaNet, which captures all dispensed drug prescriptions since January 1996 [12]. Provincial health numbers are used to link these databases at the individual-level and are then replaced by data stewards at Population Data BC with random depersonalized identifiers to maintain patient anonymity.

### Cohort

We followed a cohort of adults (≥ 18 years of age) with gout over the period of January 1, 1990 to March 31, 2018, with incident gout cases occurring between January 2, 2000 and March 19, 2018. We identified adults with gout according to the following case definition: (1) one physician visit with a principal diagnostic code for gout (*International Classification of Diseases, 9th Revision* (ICD-9): 274.x) OR one inpatient visit with principal diagnostic code for gout (*International Classification of Diseases, 10h Revision* (ICD-10): M10.x); and (2) no gout diagnosis for a 10-year run-in period before the first ICD code for gout to ensure incident gout cases are identified. The index date was defined as the first date when individuals met the case definition for gout. From a source population of one million general population controls, that is, adults (≥18 years of age) residing in the province without inflammatory arthritis (e.g., gout, rheumatoid arthritis, systemic lupus erythematosus), we matched incident gout cases to individuals without gout (1:1) based on sex, year of birth (within ± 5 years), and year of first MSP registration (within ± 5 years). Non-gout controls also had a 10-year run-in period.

### Outcome ascertainment

We applied validated case definitions to evaluate incident depression [13] (ICD-9: 296.2, 296.3, 296.5, 300.4, 309.x, and 311.x; ICD-10: F20.4, F31.3-F31.5, F32.x, F33.x, F34.1, F41.2, and F43.2) and anxiety [14, 15] (ICD-9: 300.0, 300.2; ICD-10: F40-F41) that use inpatient data from the Discharge Abstract Database and outpatient data from the MSP database. Individuals with gout and non-gout controls that met the case definition for depression or anxiety prior to the index date were excluded from their respective analyses to capture incident depression and anxiety.

### Covariate ascertainment

The covariates that we assessed in the year prior to the index date were age, sex, neighbourhood income quintile, residence (urban/rural) (Additional file 1), comorbidities as measured using the Charlson-Romano Comorbidity Index [16], and health care utilization characterized by the number of outpatient, inpatient, and rheumatology visits.

### Statistical analysis

Descriptive statistics for the study cohort were evaluated in the year prior to index date. We applied chi-square tests and t-tests to evaluate differences between individuals with a history of depression or anxiety before the gout index date to those without a prior diagnosis. We used multivariable Cox proportional hazard regression models to evaluate the onset of depression and anxiety among patients with gout in comparison to non-gout controls. Both multivariable models adjusted for the confounding effects of age, sex, neighbourhood income quintile, residence, Charlson-Romano Comorbidity Index, and health care utilization at baseline. All analyses were conducted using SAS statistical software v. 9.4 (SAS Institute, Cary, North Carolina).

### Study conduct

This study was approved by the University of British Columbia (H18-03737). All inferences, opinions, and conclusions drawn in this manuscript are those of the authors, and do not reflect the opinions or policies of the Data Steward(s).

## Results

**Figure 1:**
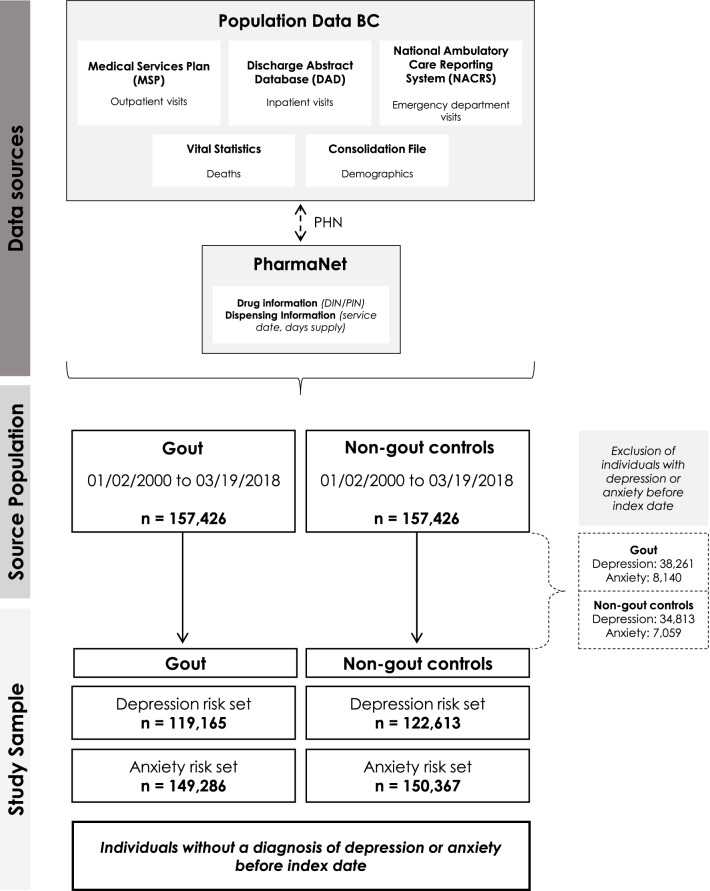
Overview of data sources, source population, and study sample (dashed arrow shows linkages between databases using provincial health numbers which are then de-identified/scrambled). DIN/PIN, drug identification number/product identification number; PHN, provincial health number

The study cohort consisted of 157,426 incident cases of gout (60.2% male; mean age 57.1 years) and 157,426 non-gout controls (60.2% male; mean age 56.9 years) (Fig. 1). Baseline characteristics are shown in Table 1.

**Table 1:** Characteristics of individuals with a gout diagnosis and comparator subjects without gout as determined in the year prior to index date

Characteristic	Gout (n = 157,426)	No gout (n = 157,426)
Age, mean (SD)	57.1 (15.7)	56.9 (15.7)
Males, n (%)	94,751 (60.2)	94,751 (60.2)
Charlson-Romano Comorbidity Index, mean (SD)	0.46 (1.11)	0.30 (0.93)
Neighbourhood income quintile, n (%)		
Quintile 1	31,252 (19.9)	29,469 (18.7)
Quintile 2	31,924 (20.3)	29,405 (18.7)
Quintile 3	34,486 (21.9)	37,794 (24.0)
Quintile 4	30,716 (19.5)	30,621 (19.5)
Quintile 5	29,048 (18.5)	30,137 (19.1)
Residence, n (%)		
Urban	135,865 (86.3)	137,493 (87.3)
Rural	21,561 (13.7)	19,933 (12.7)
Health care utilization, mean (SD)		
Number outpatient visits	11.76 (13.46)	8.13 (11.17)
Number of inpatient visits	0.30 (0.80)	0.22 (0.67)
Number rheumatology visits	0.06 (0.49)	0.04 (0.39)

Descriptive statistics were determined for the year prior to index date

SD, standard deviation

Overall, the prevalence of depression and anxiety before gout diagnosis was 24.3% (N = 38,261) and 5.2% (N = 8140), respectively. Individuals with depression or anxiety before gout diagnosis had significantly increased comorbidity index and health care utilization in the year prior to index date. In addition, a significantly higher proportion of females were diagnosed with depression or anxiety before their gout diagnosis (Table 2).

**Table 2:** Characteristics of individuals with gout according to history of depression and anxiety before their diagnosis of gout

Characteristic	Depression				Anxiety			
	With history^a^ (n = 38,261)		No history^b^ (n = 119,165)	*P* value	With history^a^ (n = 8,140)		No history^b^ (n = 149,286)	*P* value
Age, mean (SD)	57.1 (14.7)		57.1 (16.0)	0.4923	57.8 (14.9)		57.0 (15.7)	< 0.0001
Males, n (%)	17,210 (45.0)		77,541 (65.1)	< 0.0001	3,473 (42.7)		91,278 (61.1)	< 0.0001
Charlson-Romano Comorbidity Index, mean (SD)	0.60 (1.27)		0.41 (1.05)	< 0.0001	0.71 (1.38)		0.45 (1.09)	< 0.0001
Neighbourhood income quintile, n (%)								
Quintile 1	8536 (22.3)		22,716 (19.1)	< 0.0001	1882 (23.1)		29,370 (19.7)	< 0.001
Quintile 2	7668 (20.0)		24,256 (20.4)		1595 (19.6)		30,329 (20.3)	
Quintile 3	8266 (21.6)		26,220 (22.0)		1805 (22.2)		32,681 (21.9)	
Quintile 4	7222 (18.9)		23,494 (19.7)		1501 (18.4)		29,215 (19.6)	
Quintile 5	6569 (17.2)		22,479 (18.9)		1357 (16.7)		27,691 (18.6)	
Residence, n (%)								
Urban	33,162 (86.7)		102,703 (86.2)	0.0158	7106 (87.3)		128,759 (86.3)	0.0074
Rural	5099 (13.3)		16,462 (13.8)		1034 (12.7)		20,527 (13.8)	
Health care utilization, mean (SD)								
Number outpatient visits	16.70 (16.95)		10.17 (11.69)	< 0.0001	20.07 (19.87)		11.31 (12.86)	< 0.0001
Number of inpatient visits	0.42 (1.05)		0.26 (0.70)	< 0.0001	0.55 (1.25)		0.29 (0.77)	< 0.0001
Number rheumatology visits	0.09 (0.63)		0.05 (0.44)	< 0.0001	0.09 (0.63)		0.06 (0.48)	< 0.0001

Descriptive statistics were determined in the year prior to index date for gout

SD, standard deviation

^a^Individuals with gout having a history of depression or anxiety before their gout diagnosis and excluded from the risk-set

^b^Individuals with gout with no history of depression or anxiety before their gout diagnosis and included in risk-set for evaluating incident depression or anxiety

A total of 19,896 cases of incident depression were observed over a median follow-up period of 6.0 years (interquartile range [IQR] 2.7–10.4 years). The median time to depression diagnosis among individuals with gout was 3.9 years (IQR 1.7–7.0 years). The incidence rate of depression for individuals with gout was 12.9 (95% CI 12.7–13.2) per 1000 person-years and for non-gout controls was 11.1 (95% CI 10.9–11.4) per 1000 person-years. The unadjusted hazard ratio (HR) for depression when comparing individuals with a gout diagnosis to individuals without a history of gout was 1.16 (95% confidence interval [CI] 1.13–1.19). Adjustment for the confounding effects of age, sex, income, residence, comorbidities, and health care utilization, resulted in an attenuated HR of 1.08 (95% CI 1.05–1.11) (Table 3).

**Table 3:** Cox proportional hazards models for the association between gout and onset of depression and anxiety

	Depression HR (95% CI)	Anxiety HR (95% CI)
*Unadjusted model*		
Gout (vs. no gout control)	1.16 (1.13, 1.19)	1.18 (1.14, 1.23)
*Multivariable model*		
Gout (vs. no gout control)	1.08 (1.05, 1.11)	1.10 (1.05, 1.14)
Age	0.99 (0.99, 0.99)	0.99 (0.99, 0.99)
Female (vs. male)	1.56 (1.51, 1.60)	1.92 (1.85, 2.00)
Neighbourhood income quintile	0.97 (0.96, 0.98)	0.99 (0.98, 1.01)
Rural residence (vs. urban)	0.98 (0.94, 1.02)	1.01 (0.95, 1.07)
Charlson-Romano Comorbidity Index	1.03 (1.01, 1.04)	1.03 (1.01, 1.06)
Number of outpatient visits	1.02 (1.02, 1.02)	1.02 (1.02, 1.02)
Number of inpatient visits	1.04 (1.01, 1.06)	1.02 (1.00, 1.04)
Number of rheumatologist visits	1.00 (0.97, 1.03)	0.94 (0.90, 0.99)

HR, hazard ratio; 95% CI, 95% confidence interval

There were 10,483 cases of incident anxiety over a median follow-up time of 6.2 years (IQR 2.9–10.6). The median time to incident anxiety following a diagnosis of gout was 4.6 years (IQR 2.1–8.1 years). The incidence rate of anxiety was 5.4 (95% CI 5.3–5.5) per 1000 person-years among individuals with gout and 4.6 (95% CI 4.4–4.7) per 1000 person-years among non-gout controls. In comparison to non-gout controls, the unadjusted HR for anxiety for those diagnosed with gout was 1.18 (95% CI 1.14–1.23). The multivariable model that adjusted for age, sex, income, residence, comorbidities, and health care utilization, resulted in a HR of 1.10 (95% CI 1.05–1.14) (Table 3).

## Discussion

The results of our population-based cohort study indicate a modest association between gout and incident depression and anxiety. The incidence of depression following gout diagnosis as observed in our study is attenuated relative to the estimates reported by three previous studies [17, 18], the most pronounced from Singh et al. [5] (HR 1.42, 95% CI 1.38–1.45). Prior et al. [6] published the only previous study on the incidence of anxiety after gout diagnosis and reported a non-significant association (HR 1.01, 95% CI 0.87–1.16) compared to our current analysis, which showed a significant association (HR 1.10, 95% CI 1.05–1.14). Differences in sample selection and data sources are likely to have contributed to variation of estimates. Nonetheless, our study’s use of a population-based cohort capturing outpatient and inpatient visits for nearly 30 years and the application of validated case definitions for depression and anxiety strengthens our understanding of the incidence of these mental health disorders in individuals with gout.

While incident depression and anxiety are increased among patients with gout compared to controls, the respective 8% (HR 1.08, 95% CI 1.05–1.11) and 10% (HR 1.10, 95% CI 1.05–1.14) modest increases among persons with gout reported in our study may reflect the episodic nature of this chronic disease. In contrast, rheumatoid arthritis, which is often characterized by sustained disease activity, is associated with a 42–65% increased incidence of depression [19, 20]. Nonetheless, qualitative research has highlighted experiences of isolation, depression, and anxiety related to the unpredictable nature of gout flares [3]. Given that gout is predominately diagnosed in men, it is also important to understand that adherence to traditional masculine norms is associated with decreased help-seeking behaviours for mental health and different expression of depression symptoms compared to women (e.g., anger, hyperactivity, impulsivity) [21, 22]. As a result, men in our study may be misclassified as not depressed given our data source only identifies depression among those who seek mental health care. It is anticipated that this information bias is non-differential and biases our estimate towards the null. Future research that uses qualitative interviews and validated questionnaires that capture the range of gender-related symptoms can enhance our understanding of the burden of mental health disorders in this population.

Also an important consideration is how health care utilization may influence the detection and diagnosis of anxiety/depression. In our multivariable Cox proportional hazards models, time-dependent variables representing health care utilization, namely number of outpatient visits and number of inpatient visits were significantly associated with both depression and anxiety outcomes. These findings may suggest that higher frequency of encounters with the health care utilization may provide opportunities to detect and diagnose mental health conditions. Interestingly, we did not find significant associations with number of rheumatology visits and our outcomes of interest, suggesting that diagnostic opportunities are not occurring in these settings. Nonetheless, it is important that all health care providers are aware of the increased risk of depression and anxiety among patients with gout so that patients may receive appropriate support.

Our study is limited to administrative databases that only include publicly funded visits and as a consequence does not capture all health care encounters related to mental health, such as psychologist visits. We considered potential impacts of comorbidities although this was largely based on application of the Charlson-Romano Comorbidity Index and not assessment of specific conditions. However, this index includes conditions that are associated with both gout and depression (e.g., cardiovascular diseases). With our use of administrative health databases, there may be unmeasured confounding from an absence of variables that measure psychosocial determinants of health.

## Conclusions

In summary, our study shows an increased incidence of depression and anxiety following gout diagnosis, however, this is attenuated compared to previous studies in gout and other rheumatic diseases. Future research directions include using sex and gender inclusive assessments of depression and anxiety to improve its diagnosis as well as understanding the clinical implications of mental health disorders in the context of gout.

## Supplementary Information

**Additional file 1. Supplementary Table.** Conditions and corresponding International Classification of Diseases (ICD) Codes, 9th and 10th Revision used in calculating Charlson-Romano Comorbidity Index.

## Data Availability

The data that support the findings of this study are available from Population Data BC [https://www.popdata.bc.ca/] but restrictions apply to the availability of these data, which were used under license for the current study, and so are not publicly available. Data are available from Population Data BC through a data access request [dataaccess@popdata.bc.ca].

## Abbreviations

95% CI: 95% Confidence interval
HR: Hazard ratio
ICD-9: International Classification of Diseases, 9th Revision
ICD-10: International Classification of Diseases, 10th Revision
MSP: Medical services plan
