# Supplementary material for: Onset of depression and anxiety among patients with gout after diagnosis: a population-based incident cohort study
Source: BMC Rheumatol. 2022 Oct 3;6:56. doi: 10.1186/s41927-022-00288-6 (PMC9528093; doi:10.1186/s41927-022-00288-6)
Supplement: Supplementary file 1 — Additional file 1. Supplementary Table. Conditions and corresponding International Classification of Diseases (ICD) Codes, 9th and 10th Revision used in calculating Charlson-Romano Comorbidity Index. [file 41927_2022_288_MOESM1_ESM.docx]

**Supplementary Table. Conditions and corresponding International Classification of Diseases (ICD) Codes, 9^th^ and 10^th^ Revision used in calculating Charlson-Romano Comorbidity Index**

| **Condition** | **ICD9** | **ICD10** |
| --- | --- | --- |
| Myocardial Infarction | 410, 412 | I21.x, I22.x, I25.2 |
| Congestive Heart Failure | 425, 428, 429.3, 402.01, 402.11, 402.91 | I09.9,I11.0, I13.0, I13.2, I25.5, I42.0, I42.5-I42.9, I43.x, I50.x, P29.0 |
| Peripheral Vascular Disease | 440, 441, 442, 443.1, 443.2, 443.3, 443.4, 443.5, 443.6, 443.7, 443.8, 443.9, 447.1, 785.4 | I70.x, I71.x, I73.1, I73.8, I73.9, I77.1, I79.0, I79.2, K55.1, K55.8, K55.9, Z95.8, Z95.9 |
| Cerebrovascular Disease | 430, 431, 432, 433, 434, 435, 436, 437, 438, 781.4, 784.3, 997.0, 362.34 | G45.x, G46.x, H34.0, I60.x,I69.x |
| Chronic Obstructive Pulmonary Disease | 491, 492, 493, 494, 496, 415.0, 416.8, 416.9 | I27.8, I27.9, J40.x, J47.x, J60.x, J67.x, J68.4, J70.1, J70.3 |
| Dementia | 290, 331 | F00.x, F03.x, F05.1, G30.x, G31.1 |
| Connective Tissue Disease | 710, 714 | M05.x, M06.x, M31.5, M32.x, M34.x,M35.1, M35.3, M36.0 |
| Ulcer Disease | 531, 532, 533, 534 | K25.x to K28.x |
| Mild Liver Disease | 571.2, 571.5, 571.6, 571.8, 571.9 | B18.x, K70.0 to K70.3, K70.9, K71.3 to K71.5, K71.7, K73.x, K74.x, K76.0, K76.2 to K76.4, K76.8, K76.9, Z94.4 |
| Diabetes | 250.0, 250.1, 250.2, 250.3 | E10.0, E10.l, E10.6, E10.8, E10.9, E11.0, E11.1, E11.6, E11.8, E11.9, E12.0, E12.1, El2.6, E12.8, El2.9, E13.0, E13.1, E13.6, E13.8, E13.9, E14.0, E14.1, E14.6, E14.8, E14.9 |
| Hemiplegia | 342, 344 | G04.1, G11.4, G80.1, G80.2, G81.x, G82.x, G83.0 to G83.4, G83.9 |
| Moderate to Severe Renal Disease | 585, 586, V56, V420, V451 | I12.0, I13.1, N03.2 to N03.7, N05.2 to N05.7, N18.x, N19.x, N25.0, Z49.0 to Z49.2, Z94.0, Z99.2 |
| Diabetes with End Organ Damage | 250.4, 250.5, 250,6, 250,7, 250,8, 250,9 | E10.2 to E10.5, E10.7, E11.2, E11.5, E11.7, E12.2 to E12.5, E12.7,E13.2 to E13.5, E13.7, E14.2 to E14.5, E14.7 |
| Any Tumor | 140-171, 174-195, 200-208,  273.0, 273,3, V1046 | C00.x to C26.x, C30.x to C34.x, C37.x to C41.x, C43.x to C45.x, C58.x to C60.x, C76.x to C81.x, C85.x to C88.x, C90.x to C97.x |
| Moderate to Severe Liver Disease | 456.0, 456.1, 456.2, 572.2, 572.3, 572.4 | I85.0, I85.9, I86.4, I98.2, K70.4, K71.1, K72.1, K72.9, K76.5, K76.6, K76.7 |
| Metastatic Solid Tumor | 196, 197, 198, 199 | C77.x-C80.x |
| AIDS | 042, 043, 044 | B20.x-B22.x, B24.x |
